# Low Sensitivity of Abbreviated Tilt Table Testing for Diagnosing Postural Tachycardia Syndrome in Adults With ME/CFS

**DOI:** 10.3389/fped.2018.00349

**Published:** 2018-11-16

**Authors:** C. (Linda) M. C. van Campen, Peter C. Rowe, Frans C. Visser

**Affiliations:** ^1^Stichting CardioZorg, Hoofddorp, Netherlands; ^2^Department of Pediatrics, Johns Hopkins University School of Medicine, Baltimore, MD, United States

**Keywords:** postural tachycardia syndrome, orthostatic intolerance, tilt table test, myalgic encephalomyelitis, chronic fatigue syndrome

## Abstract

**Introduction:** Orthostatic intolerance is common among individuals with myalgic encephalomyelitis/chronic fatigue syndrome (ME/CFS). In some ME/CFS case definitions, orthostatic intolerance is considered a core feature of the disorder. Some studies have employed tilt table tests lasting 2–5 min to diagnose one common form of orthostatic intolerance, postural tachycardia syndrome (POTS). We examined the diagnostic yield of abbreviated durations of tilt testing in adults meeting criteria for ME/CFS, and identified the proportion with POTS misdiagnosed using testing of <10 min.

**Methods:** Eligible participants were consecutive individuals satisfying study criteria for ME/CFS and POTS evaluated at the Stichting CardioZorg (SCZ, Hoofddorp, NL) between November 2012 and August 2018. Individuals being treated with medications commonly used to manage orthostatic intolerance were excluded. Head-up tilt table testing involved 15 min of supine posture then 20 min at 70 degrees upright. Only the data from the first 10-min upright were used. POTS was defined as an increase in HR during a maximum of 10 min of upright tilt of at least 30 beats per minute (bpm), in the absence of either classical or delayed orthostatic hypotension. We measured the time until HR criteria for POTS were reached using survival curves, and compared survival curves between subgroups divided by age, sex, disease duration, and degree of hypocapnia during the test.

**Results:** Of 627 individuals with ME/CFS evaluated during the study period, 155 met criteria for POTS. The median time to reaching HR criteria for POTS was 3 min. A two-minute tilt table test would miss 55% (95% CI, 48–63%) of those meeting POTS criteria over the course of 10 min upright. The median time to reaching HR criteria for POTS did not differ by sex, age, duration of ME/CFS, or hypocapnia during tilt.

**Conclusions:** Abbreviated tilt table testing misses a substantial proportion of those ultimately diagnosed with POTS during a 10-min tilt table test, and should be abandoned for the clinical diagnosis and in epidemiologic studies designed to estimate the prevalence of POTS among those with ME/CFS.

## Introduction

Orthostatic tachycardia has been associated with clinical syndromes that resemble myalgic encephalomyelitis/chronic fatigue syndrome (ME/CFS) since at least 1940, when McLean and Allen described a group of patients who experienced excessive heart rate acceleration and hypotension after shifting from a recumbent to an upright posture (1, 2). Studies in the 1990s brought further attention to the association between ME/CFS and various forms of orthostatic hypotension or postural tachycardia syndrome (POTS) (3–8). Although neglected in the Fukuda criteria for CFS (9), and underemphasized in the ME criteria (9, 10), orthostatic intolerance is now considered a core feature in the Institute of Medicine ME/CFS criteria (11).

The various consensus criteria for POTS all require a heart rate (HR) increase of at least 30 beats per minute over the course of 10 min upright for adults (or at least 40 bpm for those 12–19 years) compared to measurements in the supine position (12–14). These HR changes can be assessed using either passive or active standing maneuvers, or head-up tilt testing (15–21). The diagnosis of POTS also requires the absence of orthostatic hypotension (OH), although the 2011 consensus criteria are somewhat unclear as to whether this refers just to OH during the first 3 min or also to delayed OH after the 3-min point. While all of the criteria mention that POTS is usually associated with chronic orthostatic symptoms, not all criteria specifically require this.

When POTS is diagnosed using the 2011 consensus criteria, the duration upright would need to be 10 min. Questions have been raised about whether a shorter period of study would be sufficient, or could identify a more impaired patient population. For example, the seminal early study by Schondorf and colleagues used a 5-min period of head-up tilt (22). Braune and colleagues have suggested that a 2-min duration of upright posture is sufficient for the diagnosis in most instances (20). A 2-min period of standing was adopted by Hoad and colleagues in defining a 27% prevalence of POTS among those with ME/CFS (23). Stewart and colleagues suggested a 5-min tilt test might suffice for diagnosing POTS in the pediatric population (14).

Roma and colleagues have recently reported that an abbreviated 2-min test would miss 53% of those who ultimately satisfy heart rate criteria for POTS over 10 min of passive standing (24). In that study of young people (median age 17 years) the median time to POTS was 3 min. Those diagnosed in the first 5 min upright had higher peak heart rates than those diagnosed in the final 5 min, and were more likely to reach a peak HR > 120 bpm. Symptom provocation during the standing test, however, was similar for the sub-groups meeting POTS criteria early vs. late in the 10 min upright.

We sought to re-examine the diagnostic yield of abbreviated orthostatic testing in a sample of patients from an adult as opposed to a primarily adolescent population with ME/CFS, using tilt testing as the form of orthostatic stress rather than passive standing.

## Materials and methods

### Eligible participants

Consecutive individuals satisfying study criteria for ME/CFS and POTS were included in this study if they had been evaluated at the SCZ (Stichting CardioZorg) between November 2012 and August 2018. The SCZ is a specialty clinic in Hoofddorp, The Netherlands. All participants had been referred by their general practitioners for either ME/CFS or orthostatic intolerance. No participants were self-referred. The use of clinical data for descriptive studies was approved by the ethics committee of the Slotervaart Hospital.

ME/CFS was considered present if participants met both the 1994 International Chronic Fatigue Syndrome Study Group criteria for CFS (25) and the 2011 international consensus definition of ME (10). Participants with ME/CFS entered the study with the expectation that they would be followed and treated clinically.

All participants were evaluated by the same experienced clinician (FV), who conducted a history and physical examination to confirm or establish the diagnosis of ME/CFS, and also conducted a tilt table test. For the tilt testing component, individuals being treated with medications that could lower heart rate or blood pressure (for example, beta-adrenergic antagonists, anti-hypertensive medications, or ivabradine) were excluded, as were those being treated with midodrine, fludrocortisone, desmopressin, pyridostigmine bromide, or stimulant medications. Individuals being treated with selective serotonin reuptake inhibitors or serotonin norepinephrine reuptake inhibitors continued to take these medications.

### Tilt test methods

Participants were studied in a climate-controlled room where the temperatures range from 22 to 24 degrees C. No intravenous or intra-arterial cannulation was employed. Nasal prongs were placed to measure expired carbon dioxide (CO2) concentrations. Individuals were positioned supine for 15 min before a motorized table brought them to a 70-degree upright position over approximately 30 s. Participants remained in the head-up tilt position for up to 20 min. The test was prematurely stopped at the request of the patient, or if the individual developed syncope or presyncope. For this study, only the data from the first 10-min upright were used. Testing was conducted at least 3 h after a light meal. Participants were encouraged to ingest an ample amount of fluid on the day of the procedure, but did not drink fluids in the 2 h before the test. Heart rate (HR), systolic, diastolic, and mean blood pressures (SBP, DBP, and MAP) were continuously recorded by finger plethysmography using the Nexfin device (BMeye, Amsterdam, NL). HR and BP data were extracted from the Nexfin device and imported into an Excel spreadsheet, and a curve-fitting procedure was used to define heart rate and BP data for the pre-test supine HR and at each discrete 1-min interval during the test (GraphPad Prism, version 6.05, GraphPad Software, La Jolla, California, USA, www.graphpad.com).

### Definition of POTS

We used the 2011 consensus definition for POTS, which requires a sustained increase in HR during a maximum of 10 min of upright tilt of at least 30 beats per minute (bpm) in those >19 years (12). The peak HR was compared to the calculated HR value at the end of the 15-min supine period. POTS was diagnosed only if there was no orthostatic hypotension (a decrease in systolic blood pressure of 20 mmHg or a decrease in diastolic blood pressure of 10 mmHg) within the first 3 min of tilt (classical OH), and additionally if there was no delayed OH during the 10 min upright.

We considered the HR criteria for POTS to have been reached when the HR during tilt first reached a 30 bpm increase from the HR at the end of the 15 min supine. We analyzed the survival curves of the time until HR criteria for POTS were reached using Graph Pad Prism. We compared survival curves between subgroups divided by age, sex, disease duration, end-tidal CO2, and serotonin reuptake inhibitor status with the Mantel-Haenszel hazard ratio, using the Mantel-Cox log rank test to determine whether differences between curves were significant. We used 95% confidence intervals (CI) for the estimates of the proportion of patients with POTS who would be missed if the test were stopped at each 1-min interval.

## Results

A total of 627 individuals with ME/CFS were evaluated at the Stichting CardioZorg during the study period. We excluded those with classical OH (*N* = 16), delayed OH (*N* = 91), vaso-vagal syncope (*N* = 6), and a normal BP and HR response to tilt (*N* = 351). We also excluded subjects whose diagnosis of POTS was based on having a HR over 120 bpm standing (*N* = 4) but who did not reach a 30 bpm HR increase. Four others were excluded because insufficient data were available (*N* = 4). No participants had a co-morbid diagnosis of diabetes mellitus, SLE, rheumatoid arthritis, or Sjogren syndrome. We did not ascertain whether participants had antibodies to adrenergic receptors or an auto-immune form of POTS. This left 155 participants who met study criteria for both ME/CFS and POTS. Table 1 shows the demographic characteristics of the study population as well as baseline pre-tilt circulatory values and highest HR and BP or lowest CO2 values during tilt. Eighteen of the 155 participants (12%) were being treated with serotonin reuptake inhibitor medications at the time of tilt testing.

**Table 1:** Demographic and circulatory characteristics of the study population^*^.

**DEMOGRAPHIC**	
Female	91%
Height	173 (8) cm
Weight	69 (15) kg
Caucasian	100%
Age (years)	33 (10)
Median duration of ME/CFS	7 years
CIRCULATORY	
Supine systolic BP	131 (15)
Supine diastolic BP	79 (8)
Supine end-tidal CO2‡	36 (4)
Supine HR	79 (14)
Peak HR during tilt	118 (20)
Peak systolic BP	125 (18)
Peak diastolic BP	85 (11)
10 min upright end-tidal CO2‡	26 (6)

* *Unless otherwise specified, these values represent mean (SD). Blood pressure and end-tidal CO2 units are mm Hg*.

‡ *End-tidal CO2 measures were available for 147/155 participants*.

**Figure 1:**
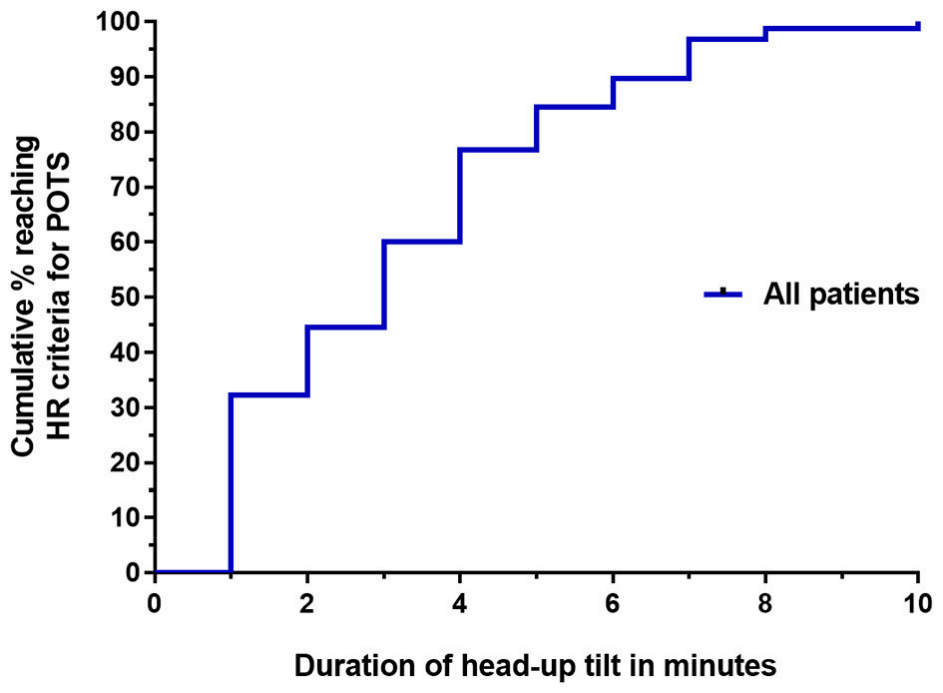
Survival curve of the time until HR criteria for POTS were reached for all 155 participants with ME/CFS.

Figure 1 illustrates the cumulative incidence of reaching HR criteria for POTS during the 10 min of upright tilt testing. The median time to reaching HR criteria for POTS was 3 min. In all participants, the HR remained elevated for the duration of the testing. Table 2 shows the proportion of participants in whom the diagnosis of POTS would have been missed if shorter durations of orthostatic stress were employed.

**Table 2:** Proportion of POTS diagnoses that would be missed at each minute of an abbreviated head-up tilt table test.

**Minutes upright**	**POTS diagnoses missed at each minute**	
	%	(95% CI)
1	68	(60–75)
2	55	(48–63)
3	40	(33–48)
4	23	(17–30)
5	15	(11–22)
6	10	(6–16)
7	3	(1–7)
8	2	(0–4)
9	2	(0–4)
10	0	

**Figure 2:**
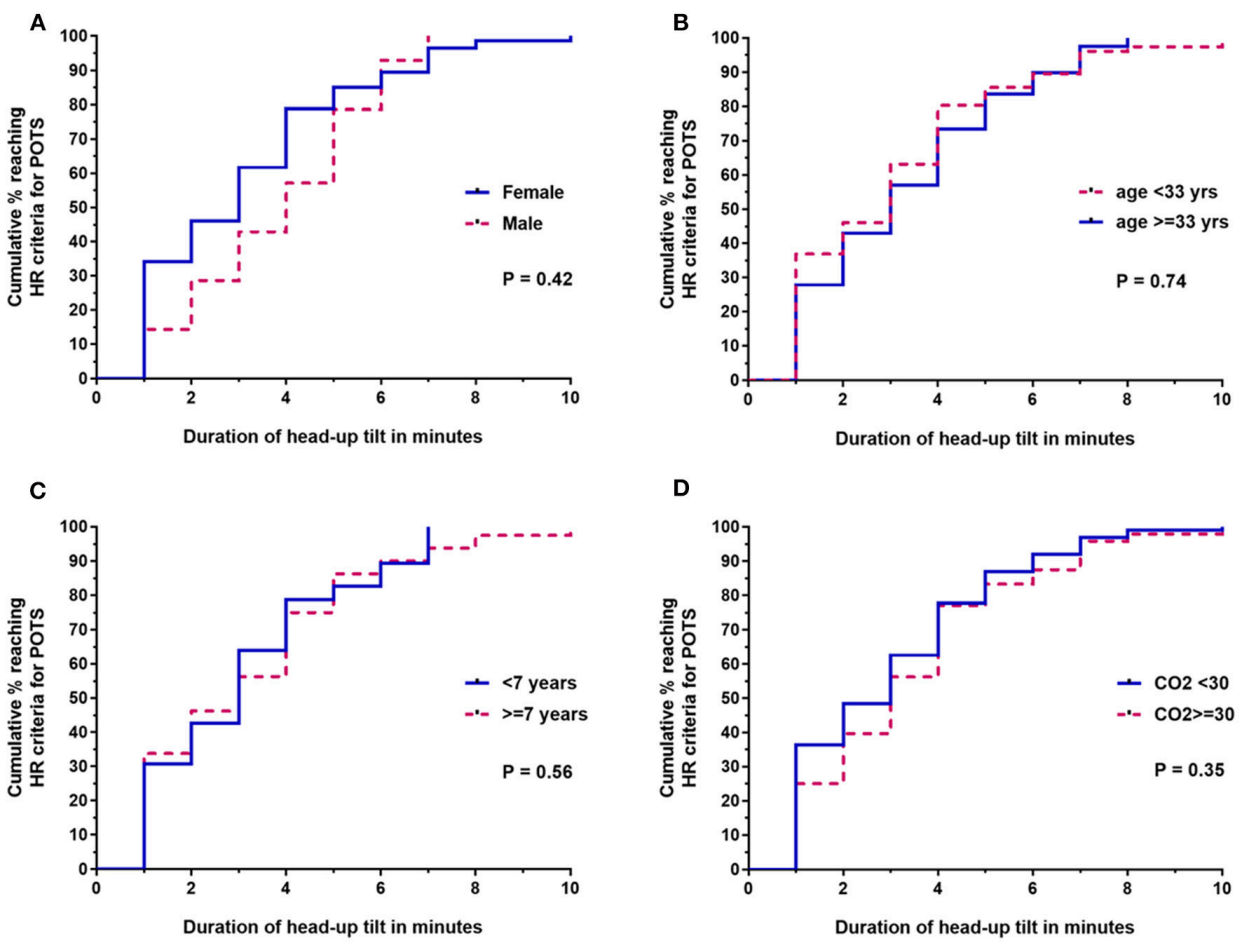
Survival curve comparisons for the time until HR criteria were reached for ME/CFS subsets based on **(A)** sex, **(B)** age (<33 vs. ≥33 years), **(C)** duration of ME/CFS at the time of tilt (<7 vs. ≥7 years), and **(D)** lowest end-tidal CO2 during tilt (<30 vs. ≥30 mm Hg). End-tidal CO2 data were available for 147/155 participants; all other analyses were based on the entire sample of 155.

Figures 2A–D shows the survival curve differences by sex, age, duration of disease, and end tidal CO2 values, respectively. The median time to reaching HR criteria for POTS was 3 min for females and 4 min for males (Mantel-Haenszel ratio, 1.3; 95% CI, 0.69–2.41; *P* = 0.42). Using a cut-point for age at the median age of our study population, there was no difference in the time to reaching HR criteria for POTS by those under 33 vs. 33 or older (Mantel-Haenszel ratio, 1.1; 95% CI, 0.72–1.58; *P* = 0.74). Using a cut-point for disease duration at the median for participants in the study, there was no difference in the time to reaching HR criteria for POTS among subjects with a disease duration of <7 years vs. more than 7 years (Mantel-Haenszel ratio, 1.12; 95% CI, 0.76–1.66; *P* = 0.56). There was no difference in the time to reaching HR criteria for POTS among subjects with an end-tidal CO2 level at the end of 10 min of <30 vs. 30 mm Hg or more (Mantel-Haenszel ratio, 1.22; 95% CI 0.89–1.85; *P* = 0.28). There was no difference in the time to reaching HR criteria for POTS for those being treated with serotonin reuptake inhibitors compared to those not being treated (Mantel-Haenszel ratio, 0.89; 95% CI, 0.0.50–1.59; *P* = 0.69).

One hundred thirty-one (85%) met HR criteria for POTS during the first 5 min upright vs. 24 (15%) in the last 5 min upright. The proportion with a peak HR of ≥ 120 was 47% in the early group and 21% in the late group (*P* = 0.02, Fisher's exact test).

## Discussion

The main finding of this study is that for adults with ME/CFS, abbreviated tilt table testing has the potential to miss a substantial proportion of those ultimately diagnosed with POTS during a 10-min tilt table test. A two-minute test would miss 55% (95% CI, 48–63%), emphasizing the limitations of POTS prevalence estimates based on abbreviated orthostatic testing. The median time to reaching HR criteria for POTS during tilt testing was 3 min, identical to the findings of Roma and colleagues in younger individuals who were studied using a passive standing test (24). We found no differences in the time to reaching the HR criteria for POTS based on age, sex, duration of ME/CFS, or the end-tidal CO2 levels at the end of the 10 min upright.

Other studies have examined the optimal time to tilt testing for the diagnosis of POTS (26). In 28 patients selected for tilt testing based on a suspicion of POTS, but in whom the ME/CFS status was not reported, Carew and colleagues reported that all 28 meeting HR criteria for POTS had done so by 7 min of 70-degree tilt, whereas none of 28 age-matched controls had developed a sustained tachycardia (26). While our study identified an additional 3% with POTS after the 7-min point, this minor variability in outcomes is likely related to the larger sample size in our study. Carew and colleagues concluded that the full 10 min of tilt was required to diagnose POTS.

The time to reaching HR criteria for POTS in our study among adults was the same as that for a primarily adolescent and young adult population. However, the proportion with a peak HR of 120 bpm or higher in the first 5 min of tilt was 47%, which compares to just 26% in the Roma study (24). Plash and colleagues reported a mean HR increase for those with POTS that was five beats higher after 5 min of upright tilt than after the same period of active standing, although this difference did not reach statistical significance until participants with POTS and healthy controls were combined (17). In their combined study population, a higher HR during passive tilt vs. active standing was present for all 5-min intervals of the 30 min of upright posture. A potential contributor to this difference, among other physiologic changes, is that active standing involves greater use of the leg muscles, more postural sway than tilt, and greater engagement of the abdominal muscles, all of which can combine to increase venous return to the heart (17). The “passive” element of a passive standing test involves leaning back against a wall, which reduces postural sway, but the leg and abdominal muscles are nonetheless expected to be more actively engaged while standing than during upright tilt. As a result, we believe the more robust HR changes in the first 5 min of tilt testing in this study compared to that of Roma and colleagues could be related in part to differences between the forms of orthostatic testing in the two studies. Other important differences such as age and disease duration could also play a role.

Among the strengths of this study are its relatively large sample size, the lack of variability in the application of diagnostic criteria due to a single examiner, a consistent tilt testing protocol, and the fact that participants satisfied the definitions for both ME and CFS. One limitation is that the study may not be applicable to all individuals with POTS, as the study enrolled only those with POTS and ME/CFS. Adults meeting case definitions for both ME/CFS and POTS report a significantly greater prevalence of severe fatigue, unrefreshing sleep, muscle pain, post exertional fatigue, and headaches than those with POTS alone (27). Similar observations of increased symptom burden for those with CFS and POTS vs. POTS alone results have been found in pediatric patients (8). Without data on time to reaching HR criteria for those with POTS in the absence of ME/CFS, we are unsure what effect the differences in general symptom burden would have on our results, and we do not have data to suggest that our findings can be extrapolated with confidence to those with POTS alone.

All individuals in this study had chronic orthostatic symptoms. The proportion of individuals with ME/CFS who have exaggerated postural tachycardia during tilt in the absence of chronic orthostatic intolerance symptoms is difficult to determine. All those with ME/CFS have chronic symptoms of fatigue and exercise intolerance, which are viewed in the autonomic literature to be features consistent with orthostatic intolerance. A high proportion with ME/CFS have lightheadedness, 96% in some studies (5), and 61% report intolerance of being on their feet (28). Across several studies, over 90% with ME/CFS report cognitive dysfunction, but in most ME/CFS studies, little effort is made to distinguish whether these symptoms are a consequence of orthostatic stress or due to some other contributor to ME/CFS pathophysiology. Some of the variability in the reporting of orthostatic symptoms in the ME/CFS literature is due to differences in the comprehensiveness with which orthostatic intolerance symptoms are ascertained (11). We are not aware of data reporting an exaggerated tachycardia among those with ME/CFS in the absence of chronic orthostatic symptoms.

We did not perform more extensive autonomic testing using quantitative sudomotor axon reflex testing, Valsalva, or heart rate variability measures to identify specific pathophysiologic subgroups of those with POTS, nor did we evaluate for small fiber neuropathy. Our focus was to determine whether abbreviated testing would miss a high proportion of those meeting the current definitions for POTS. Future studies will be able to examine whether specific POTS subgroups have different heart rate responses to upright tilt, and whether healthy controls have a parallel HR elevation at different points of the tilt test. Future studies with healthy controls will also be able to ascertain whether the interaction between time and HR elevation is similar in healthy individuals.

Participants in this study were not being treated with medications typically used to directly modulate heart rate and blood pressure, and only 18 individuals (12%) were being treated with SSRI or SNRI medications at the time of tilt testing. The survival curves of the time to reaching HR criteria for POTS, however, did not differ between those on SSRI/SNRI medications and those not being treated, suggesting that medication status did not affect the results. We acknowledge that serotonin has the potential to affect vascular tone (both vasoconstriction and vasodilation) (29), and the SNRI medications in the clinical setting can raise BP, but our data suggest a limited or absent impact on the detection of POTS among those with ME/CFS. The impact of these medications on other forms of orthostatic intolerance remains to be determined.

We conclude that abbreviated orthostatic testing should be abandoned in epidemiologic and clinical studies designed to estimate the prevalence of POTS among those with ME/CFS, in whom a full 10-min of tilt testing improves the sensitivity of the test for identifying POTS.

## Data availability statement

The raw data supporting the conclusions of this manuscript will be made available by the authors, without undue reservation, to any qualified researcher.

## Author contributions

CvC, PR, and FV conceived the study, CvC and FV collected the data, CvC performed the primary data analysis and FV and PR performed secondary data analyses. All authors were involved in the drafting and review of the manuscript.

### Conflict of interest statement

The authors declare that the research was conducted in the absence of any commercial or financial relationships that could be construed as a potential conflict of interest.
